# Low-Grade Endotoxemia and Thrombosis in COVID-19

**DOI:** 10.14309/ctg.0000000000000348

**Published:** 2021-06-04

**Authors:** Alessandra Oliva, Vittoria Cammisotto, Roberto Cangemi, Domenico Ferro, Maria Claudia Miele, Massimiliano De Angelis, Francesca Cancelli, Pasquale Pignatelli, Mario Venditti, Francesco Pugliese, Claudio Maria Mastroianni, Francesco Violi

**Affiliations:** 1 Department of Public Health and Infectious Diseases, Sapienza University of Rome, Rome, Italy;; 2 Department of General Surgery and Surgical Speciality Paride Stefanini, Sapienza University of Rome, Rome, Italy;; 3 Department of Translational and Precision Medicine, Sapienza University of Rome, Rome, Italy;; 4 Department of Clinical Internal, Anesthesiologic and Cardiovascular Sciences, Sapienza University of Rome, Rome, Italy;; 5 Mediterranea Cardiocentro, Naples, Italy.

## Abstract

**INTRODUCTION:**

Patients with community-acquired pneumonia display enhanced levels of lipopolysaccharides (LPS) compared with controls, suggesting that low-grade endotoxemia may be implicated in vascular disturbances. It is unknown whether this occurs in patients with coronavirus 2019 (COVID-19) and its impact on thrombotic complications.

**METHODS:**

We measured serum levels of zonulin, a marker of gut permeability, LPS, and D-dimer in 81 patients with COVID-19 and 81 healthy subjects; the occurrence of thrombotic events in COVID-19 during the intrahospital stay was registered.

**RESULTS:**

Serum LPS and zonulin were higher in patients with COVID-19 than in control subjects and, in COVID-19, significantly correlated (*R* = 0.513; *P* < 0.001). Among the 81 patients with COVID-19, 11 (14%) experienced thrombotic events in the arterial (n = 5) and venous circulation (n = 6) during a median follow-up of 18 days (interquartile range 11–27 days). A logistic regression analysis showed that LPS (*P* = 0.024) and D-dimer (*P* = 0.041) independently predicted thrombotic events.

**DISCUSSION:**

The study reports that low-grade endotoxemia is detectable in patients with COVID-19 and is associated with thrombotic events. The coexistence of low-grade endotoxemia with enhanced levels of zonulin may suggest enhanced gut permeability as an underlying mechanism.

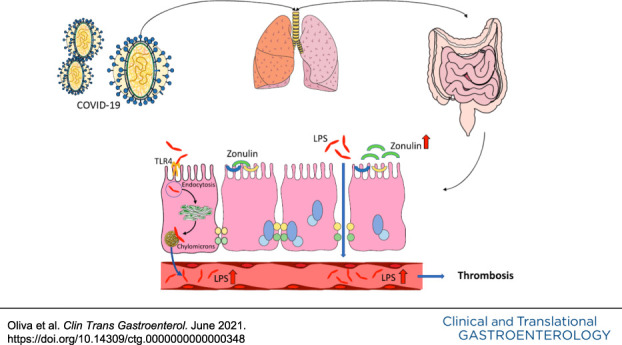

## INTRODUCTION

Coronavirus 2019 (COVID-19) is a serious pandemic characterized by severe acute respiratory syndrome (SARS-CoV-2) needing mechanical ventilation and intensive care unit treatment. Among the factors predisposing to poor survival, thrombotic complications have been suggested to have an important role. Accordingly, clinical studies showed a high prevalence of arterial and venous thromboembolism during the intrahospital stay, which independently increased the mortality risk (1,2). The coexistent coagulopathy, which is essentially characterized by enhanced levels of D-dimer, is a crucial determinant for thrombosis predisposition; however, the factors favoring clotting activation in SARS-CoV-2 have not been clarified.

Previous studies reported that patients with community-acquired pneumonia (CAP) display enhanced levels of lipopolysaccharides (LPS) coincidentally with an ongoing prothrombotic state, suggesting that low-grade endotoxemia may be implicated in the thrombotic complications occurring in CAP (3,4). This hypothesis has been recently corroborated in an experimental model of thrombosis where low-grade endotoxemia enhanced thrombus growth through Toll-like receptor 4 (TLR4) (5). Based on this, we analyzed the interplay between endotoxemia and thrombotic events in SARS-CoV-2 patients followed-up during the intrahospital stay; furthermore, we investigated whether changes in gut permeability may account for low-grade endotoxemia.

## METHODS

We included in the study adult (≥18 years) patients with laboratory-confirmed COVID-19 and SARS-CoV-2–related pneumonia, requiring or not mechanical ventilation, consecutively hospitalized in March 2020. COVID-19 was diagnosed on the basis of the World Health Organization interim guidance, as previously described (6). Patients matched for demographic and clinical characteristics but without acute infections were used as control; they were recruited as outpatients from the Division of I Clinica Medica, Atherothrombosis center, Policlinico Umberto I, Rome.

### Baseline assessment

Demographic and anamnestic characteristics, along with baseline clinical, laboratory, and radiological results, were extracted from electronic medical records of patients enrolled. Laboratory assessments consisted of blood count, coagulation tests (D-dimer, prothrombin time, activated partial thromboplastin time, and fibrinogen), assessment of liver and renal function, and routine analyses including electrolytes, high-sensitivity C-reactive protein (hs-CRP), procalcitonin, lactate dehydrogenase, and creatine kinase. Data regarding demographic characteristics, comorbidities, and concurrent therapy were collected. Pre-existence of diabetes mellitus, hypertension, cardiovascular disease, chronic kidney disease, and obesity is defined as previously described (7).

### Serum and plasma LPS assay

LPS levels in human serum and plasma were measured using a commercial ELISA kit as previously described (5). Values were expressed as picograms per milliliter; intra-assay and inter-assay coefficients of variation were <10%.

### Plasma LPS-binding protein assay

To assess whether analysis of LPS in plasma may influence the results, LPS (ELISA assay as above reported) and human LPS-binding protein (LBP; ELISA kit RayBio, Norcross, GA) were measured in patients with COVID-19 (n = 21) and healthy subjects (HS; n = 22). Demographic and clinical characteristics of patients with COVID-19 and HS are available in Supplementary Table 1 (see Supplementary Digital Content 1, http://links.lww.com/CTG/A637).

LBP values were expressed as micrograms per milliliter; intra-assay and inter-assay coefficients of variation were <10% and <12%, respectively.

### Serum zonulin assay

Serum zonulin levels were measured using a commercially ELISA kit (Elabscience, Houston, TX). The amount of zonulin was measured with a microplate autoreader at 450 nm. Values were expressed as nanograms per milliliter; both intra-assay and inter-assay coefficients of variation were <10%.

### Assessment of intrahospital ischemic and embolic events

The clinical course of the disease and its evolution was monitored during hospitalization. The appearance of new ischemic/embolic events was diagnosed as follows: (i) pulmonary thromboembolism by lung computed tomography scan (8); (ii) myocardial infarction by EKG changes associated with enhanced markers of cell necrosis (9); (iii) acute brain ischemia by onset of new focal neurological signs and symptoms and confirmed, whenever possible, by nuclear magnetic resonance or computed tomography imaging (10); and (iv) acute limb ischemia diagnosed according to American Heart Association guidelines (11).

### *Ex vivo* study

#### Platelet preparation

To obtain platelet-rich plasma, 3.8% sodium citrate sample from patients with COVID-19 (n = 21) and HS (n = 22) was centrifuged for 15 minutes at 180*g*. After, samples were centrifuged for 3 minutes at 3,000 rpm, and pellets were stored at −80 °C for western blotting analysis of Toll/interleukin-1 receptor domain-containing adapter protein (TIRAP) and TIRAP phosphorylation (p-TIRAP) and detection of TYRAP/Myeloid differentiation factor 88 (MyD88) complex.

### Co-immunoprecipitation assays and western blot analysis

Co-immunoprecipitation assays were performed in platelet pellets obtained from patients with COVID-19 and HS. Cells were suspended in radioimmunoprecipitation assay buffer with protease and phosphatase inhibitors cocktail (10 μg/mL; Thermo Fisher Scientific, Waltham, MA) and incubated on ice for 30 minutes. The protein concentration was determined by Bradford assay, and the cellular proteins (500 μg) were incubated with an anti-mouse monoclonal antibody anti-MyD88 (Santa Cruz Biotechnology, Dallas, TX) or anti-rabbit polyclonal antibody anti-TIRAP (Invitrogen, Carlsbad, CA) (1-μg/mg protein) overnight at 4 °C. Then, protein-A/G agarose (Santa Cruz Biotechnology) was added to the mixtures and adequately resuspended. After incubation for 60 minutes in ice, the mixtures were centrifuged and pellet washed with radioimmunoprecipitation assay buffer. Finally, the immunoprecipitates were dissolved in lysis buffer containing 2X Leammli sample buffer and 20% of 2-mercaptoethanol (Bio-Rad, Hercules, CA). Proteins were separated by SDS-PAGE on 10% polyacrylamide gel and then transferred to nitrocellulose membranes. Finally, the membranes were blocked with bovine serum albumin (5%) and incubated overnight at 4 °C with anti-rabbit polyclonal antibody anti-TIRAP or anti-mouse monoclonal antibody anti-MyD88.

To determinate p-TIRAP, we used procedure described above, but, immediately after cell lysis, 30 μg/lane was separated by SDS-PAGE on 12% polyacrylamide gel. Moreover, membranes were incubated with rabbit polyclonal antibody anti-TIRAP and anti-p-TIRAP (Thermo Fisher Scientific) overnight at 4 °C. Then, the membranes were incubated with secondary antibody (1:3000; Bio-Rad), and the co-immune and immune complexes were detected by increased chemiluminescence substrate (ECL Substrates; Bio-Rad). Densitometric analysis of the bands was performed using ImageJ software.

## RESULTS

Clinical characteristics of patients with COVID-19 and controls are reported in Table 1 and in Supplementary Table 1 (see Supplementary Digital Content 1, http://links.lww.com/CTG/A637). No significant differences were present for age, sex, body mass index, smoking habit and prevalence of arterial hypertension, diabetes, and atrial fibrillation between patients and controls; coronary heart disease, heart failure, and chronic obstructive pulmonary disease showed a nonsignificant tendency to be more prevalent among patients with COVID-19.

**Table 1.** Clinical characteristics of patients with COVID-19 and control subjects

	Control subjects	Patients with COVID-19	*P*
N	81	81	
Age (yr)	64.5 ± 6.9	64.1 ± 14.0	0.823
BMI (kg/m^2^)	26.1 ± 3.5	27.5 ± 1.9	0.389
Male sex	60%	60%	1.000
Arterial hypertension	47%	42%	0.635
Smokers	15%	11%	0.641
COPD	9%	17%	0.159
Diabetes	16%	14%	0.328
CAD	7%	15%	0.210
Heart failure	2%	9%	0.167
Atrial fibrillation	6%	10%	0.471
ACE inhibitors/ARBs	40%	35%	0.427
hs-CRP	—	4.8 [1.7–13.9]	n.a.
D-dimer	—	1,210 [1,210–2,771]	n.a.
LPS (pg/mL)	13 [6–29]	50 [23–73]	<0.001
Zonulin (ng/mL)	1.9 [1.2–2.2]	2.9 [2.0–3.7]	<0.001

Differences between percentages were assessed by Fisher exact tests. All continuous variables were tested for normality with the Shapiro-Wilk test. The Student unpaired *t* test was used for normally distributed continuous variables (expressed as mean ± SD). The Mann-Whitney *U* test was used for non-normally distributed continuous variables (expressed as median [interquartile range]).

ACE, angiotensin-converting enzyme; ARB, angiotensin receptor blocker; BMI, body mass index; CAD, coronary heart disease; COPD, chronic obstructive pulmonary disease; COVID-19, coronavirus 2019; hs-CRP: high-sensitivity C-reactive protein; LPS, lipopolysaccharides; n.a., not applicable.

**Figure 1.**
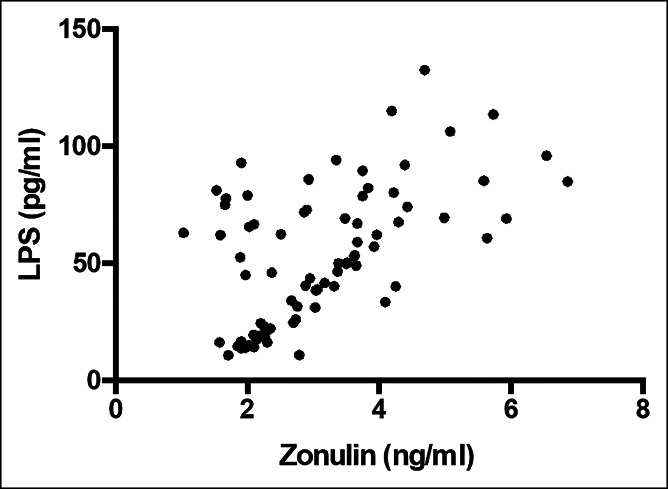
Scatter plot showing significant (2-tailed) Spearman positive correlation of zonulin in horizontal vs vertical directions of LPS concentration.

Serum LPS and zonulin were higher in patients with COVID-19 than in control subjects (Table 1); in COVID-19, LPS significantly correlated with zonulin (Figure 1) (*R*s = 0.513; *P* < 0.001) and hs-CRP (*R*s = 0.544; *P* < 0.001). A linear regression model showed that after log-transformation LPS correlated with zonulin without a significant effect of age or baseline comorbidities (not shown). Furthermore, analysis repeated in plasma of a subgroup of COVID-19 and HS showed that both LPS (50.2 [26.2–66.9] vs 12.0 [7.8–19.6] pg/mL; *P* < 0.001) and LBP (20.28 [13.29–25.61] vs 14.34 [13.12–14.92] μg/mL; *P* = 0.048) were higher in patients compared with controls and significantly correlated (*R*s = 0.684; *P* < 0.001).

Among the 81 patients with COVID-19, 11 patients (14%) experienced thrombotic events in the arterial (n = 5) and venous circulation (n = 6) during a median follow-up of 18 days (interquartile range 11–27 days).

Clinical characteristics of patients who experienced or not a thrombotic event are reported in Table 2. No significant differences were found in baseline clinical characteristics between the 2 groups. Patients who experienced thrombotic events showed higher levels of hs-CRP, D-dimer, and LPS. A logistic regression analysis, aimed to estimate the effect of these biomarkers on thrombotic events, showed that LPS (odds ratio 2.575; 95% confidence interval 1.135–5.845; *P* = 0.024; for each increasing quartile) and D-dimer (odds ratio 2.223; 95% confidence interval 1.033–4.782; *P* = 0.041, for each increasing quartile) independently predicted thrombotic events.

**Table 2.** Clinical characteristics of patients with COVID-19 who experienced or not a thrombotic event during the in-hospital stay

	Patients without thrombotic events	Patients with thrombotic events	*P*
N	70	11	
Age (yr)	63.2 ± 14.2	68.8 ± 13.2	0.226
BMI (kg/m^2^)	27.5 ± 1.9	27.8 ± 2.2	0.897
Male sex	57%	82%	0.186
Arterial hypertension	42%	45%	1.000
Smokers	7%	18%	0.240
COPD	15%	33%	0.189
Diabetes	16%	0%	0.347
CAD	14%	18%	0.649
Heart failure	6%	18%	0.198
Atrial fibrillation	9%	9%	1.000
ACE inhibitors/ARBs	33%	33%	1.000
hs-CRP	3.8 [1.2–10.8]	20.3 [4.8–28.3]	0.003
D-dimer	1,180 [672–2,194]	3,913 [1,713–4,505]	0.010
LPS (pg/mL)	45 [21–69]	79 [62–96]	0.003
Zonulin (ng/mL)	2.8 [2.1–3.7]	3.7 [2.1–4.4]	0.247

Differences between percentages were assessed by Fisher exact tests. All continuous variables were tested for normality with the Shapiro-Wilk test. The Student unpaired *t* test was used for normally distributed continuous variables (expressed as mean ± SD). The Mann-Whitney *U* test was used for non-normally distributed continuous variables (expressed as median [interquartile range]).

ACE, angiotensin-converting enzyme; ARB, angiotensin receptor blocker; BMI, body mass index; CAD, coronary heart disease; COPD, chronic obstructive pulmonary disease; COVID-19, coronavirus 2019; hs-CRP, high-sensitivity C-reactive protein; LPS, lipopolysaccharides.

### In *ex vivo* study

**Figure 2.**
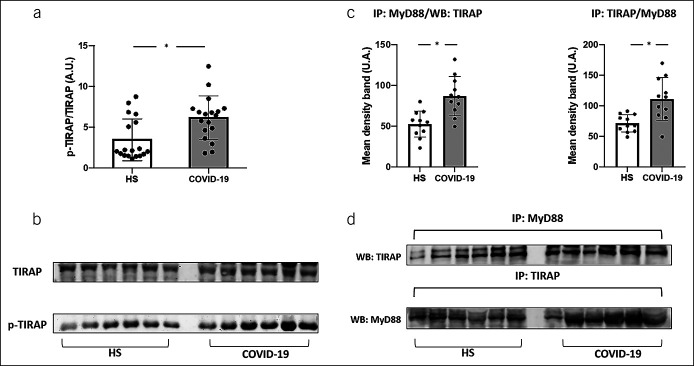
TIRAP phosphorylation (n = 21, **a**), MyD88 and TIRAP co-immunoprecipitation (n = 12, **c**) in patients with COVID-19 and HS. Data are expressed as mean values ± SDs and *P < 0.05. Representative western blot bands of TIRAP phosphorylation and immune complexes TIRAP/MyD88 (**b** and **d**). TIRAP, Toll/interleukin-1 receptor domain-containing adapter protein. A.U., arbitrary unit; HS, Healthy subjects; MyD88, Myeloid differentiation factor 88.

To examine the role of LPS on platelet activation in patients with COVID-19, we evaluated the phosphorylation of TIRAP, that is marker of TLR4 activation (12). We observed that platelets from patients with COVID-19 showed a greater phosphorylation of TIRAP than HS (Figure 2, panels a and b). Furthermore, the immunocomplex formation of TIRAP with MyD88, called myddosome, that is required for TLR4 signal transduction (12), was significantly higher in platelets from patients with COVID-19 compared with HS (Figure 2, panels c and d).

### Statistical analysis

Continuous variables were assessed for normality with the Shapiro-Wilk test. Variables with normal distribution were expressed as mean values and SDs sand tested for differences using a *t* test. Non-Gaussian variables were expressed as median and interquartile range, differences were tested using the Mann-Whitney test, and correlations were analyzed by the Spearman rank correlation test. Categorical variables were expressed as percentages and analyzed by the χ^2^ test. Linear regression models were used to estimate effects on continuous outcomes. The bivariate and multivariate effects of prognostic factors on thrombotic events were assessed by means of logistic regression models.

Only *P* values <0.05 were considered statistically significant. All tests were 2-tailed, and analyses were performed using computer software packages (SPSS Statistics 25; IBM, Armonk, NY).

## DISCUSSION

The study reports for the first time that low-grade endotoxemia is detectable in patients with COVID-19 complicated by thrombotic events. The association between low-grade endotoxemia with enhanced gut permeability suggests intestinal tube functionality changes as a mechanism accounting for vascular damage.

About 100 trillion of gut bacteria contribute to an enteric reservoir of >1-g LPS, which is detected in the blood of healthy subjects in a range of roughly 1–30 pg/mL (13). We have previously reported that low-grade endotoxemia is detectable in patients with CAP, but the impact of this change with thrombosis was not investigated (14). Here, we report that COVID-19 displays elevated levels of LPS, which are significantly associated with intrahospital thrombotic complications, suggesting that LPS may promote an ongoing prothrombotic state. The difference between patients and controls is not biased by concomitant atherosclerotic risk factors or cardiac disease such as atrial fibrillation, which, in fact, were equally distributed between the 2 groups.

The biological plausibility of this finding relies on previous experimental study showing that low-grade endotoxemia amplifies thrombus growth through interaction with its receptor TLR4 at level of platelets or leucocytes (15). This finding is in agreement with previous reports showing that gut microbiota enhances thrombus growth through TLR2-dependent platelet activation (16,17). At this regard, Shin et al. demonstrated *in vitro* and *in vivo* that bacterial lipoproteins promote thrombosis by clotting activation and endothelial leakage, which is a typical feature of COVID-19 (18). To further explore whether TLR4 receptor is actually activated in COVID-19, in a subgroup of patients and controls, we measured the intracellular TIRAP, also known as Mal (MyD88 adaptor-like), which participates in TLR4 signal transduction where it acts as an adapter for MyD88 recruitment (19); thus, tyrosine phosphorylation of TIRAP is required for adapter signaling and regulates TIRAP interaction with TLR4 and receptor signaling (12). Even if these findings are in favor of TLR4 activation by LPS, we cannot exclude that the receptor may be activated by molecules other than TLR4 such as oxidized LDL or saturated fatty acids (20,21).

Alternatively, LPS may indirectly promote thrombosis by enhancing the production of inflammatory cytokines, such as tumor necrosis factor α, that is a proaggregating molecule (22); it is of interest, at this regard, the significant association between LPS and CRP.

The increased concentration of LPS in the peripheral circulation of patients with COVID permeability may be an intriguing possibility accounting for LPS translocation into systemic circulation and eventually systemic inflammation. Thus, LPS translocation into systemic circulation activates macrophages, so favoring inflammation of visceral adipose tissue and hepatic Kupfer cells resulting in nonalcoholic fatty liver disease (23). We have previously reported that patients with myocardial infarction and liver disease display high systemic levels of zonulin, which modulates gut permeability by disassembling the intercellular tight junctions (24). Experimental and clinical studies demonstrated that zonulin upregulation plays a role in increasing gut permeability and that serum levels of zonulin correlate with enhanced intestinal permeability (25). However, we must acknowledge the uncertainty regarding the validity of our zonulin assay (26); therefore, direct analysis of gut permeability such as the intake of lactose-mannitol is necessary to confirm our hypothesis (27). Furthermore, we cannot exclude that the elevation of LPS may depend on other mechanisms including enhanced endocytosis or chylomicron uptake or pneumonia-related bacteremia, as evidenced in patients with CAP (28,29).

The study has several limitations. We did not explore the mechanisms accounting for enhanced gut permeability; however, previous study showed that COVID-19 binding and entry into human cells occurs through converting enzyme 2, which localizes not only in the lung but also in the gastrointestinal tube (30).

We did not find an association between LPS and D-dimer, which is a marker of clotting activation; this does not exclude, however, that LPS behave as a prothrombotic molecule because it acts at level of cell signaling that could not be intercepted by D-dimer.

In conclusion, low-grade endotoxemia coexists and is associated with thrombotic events in COVID-19, providing a new insight into the mechanism eliciting inflammation and eventually thrombosis in COVID-19. Further study is necessary to investigate whether changes of gut permeability play a role in eliciting low-grade endotoxemia.

## CONFLICTS OF INTEREST

**Guarantor of the article:** Francesco Violi, MD.

**Specific author contributions:** Alessandra Oliva, MD, and Vittoria Cammisotto, MSc, equally contributed. A.O.: study design and coordination. V.C.: laboratory analysis. R.C.: statistical analysis and draft elaboration. D.F.: patients' recruitment and follow-up. M.C.M., M.D.A., and F.C.: laboratory analysis. P.P.:

**Study Highlights**

**WHAT IS KNOWN**

✓ Low-grade endotoxemia plays a role in thrombotic complications in patients with coronavirus 2019 (COVID-19).

**WHAT IS NEW HERE**

✓ Serum zonulin and lipopolysaccharide levels are higher in patients with COVID-19.
✓ Higher gut permeability and low-grade endotoxemia are associated with thrombosis.

**TRANSLATIONAL IMPACT**

✓ Gut permeability modulation may help to counteract vascular damage in COVID-19.

data interpretation and draft elaboration. M.V., F.P., and C.M.M.: data interpretation and article elaboration. F.V.: study conception and coordination, data interpretation, and writing the manuscript.

**Financial support:** This research was funded by grant from PhD course: Fisiopatologia Ed Imaging Cardio-Toraco-Vascolare, Sapienza University of Rome.

**Potential competing interests:** None to report.

## Supplementary Material

SUPPLEMENTARY MATERIAL
