# Supplementary material for: Low-Grade Endotoxemia and Thrombosis in COVID-19
Source: Clin Transl Gastroenterol. 2021 Jun 4;12(6):e00348. doi: 10.14309/ctg.0000000000000348 (PMC8183715; doi:10.14309/ctg.0000000000000348)
Supplement: SUPPLEMENTARY MATERIAL [file ct9-12-e00348-s001.docx]

|  | **Healthy Subjects (HS)** | **COVID-19 patients** | **P value** |
| --- | --- | --- | --- |
| **N.** | 22 | 21 | - |
| **Age (years)** | 59.3±7.3 | 63.3±16.2 | 0.704 |
| **BMI (kg/m^2^)** | 21.7 ±2.2 | 26.7±3.1 | 0.085 |
| **Gender** | F 18, M= 4 | F 21, M 0 | 0.082 |
| **Arterial hypertension** | 0% | 30% | 0.002 |
| **Smokers** | 12% | 10% | 0.962 |
| **COPD** | 0% | 5% | 0.323 |
| **Diabetes** | 0% | 15% | 0.075 |
| **CAD** | 0% | 10% | 0.154 |
| **Heart failure** | 0% | 5% | 0.323 |
| **Atrial fibrillation** | 0% | 5% | 0.323 |
| **ACE-inhibitors/ARBs** | 0% | 30% | 0.002 |
